# Estimating population density of insectivorous bats based on stationary acoustic detectors: A case study

**DOI:** 10.1002/ece3.5928

**Published:** 2020-01-28

**Authors:** Markus Milchram, Marcela Suarez‐Rubio, Annika Schröder, Alexander Bruckner

**Affiliations:** ^1^ Institute of Zoology Department of Integrative Biology and Biodiversity Research University of Natural Resources and Life Sciences Vienna Vienna Austria

**Keywords:** acoustic monitoring, automated recording units, Chiroptera, environmental assessment, generalized random encounter models, population density, Royle–Nichols models, temperate forest

## Abstract

1. Automated recording units are commonly used by consultants to assess environmental impacts and to monitor animal populations. Although estimating population density of bats using stationary acoustic detectors is key for evaluating environmental impacts, estimating densities from call activity data is only possible through recently developed numerical methods, as the recognition of calling individuals is impossible.
2. We tested the applicability of generalized random encounter models (gREMs) for determining population densities of three bat species (Common pipistrelle *Pipistrellus pipistrellus*, Northern bat *Eptesicus nilssonii*, and Natterer's bat *Myotis nattereri*) based on passively collected acoustical data. To validate the results, we compared them to (a) density estimates from the literature and to (b) Royle–Nichols (RN) models of detection/nondetection data.
3. Our estimates for *M. nattereri* matched both the published data and RN‐model results. For *E. nilssonii*, the gREM yielded similar estimates to the RN‐models, but the published estimates were more than twice as high. This discrepancy might be because the high‐altitude flight of *E. nilssonii* is not accounted for in gREMs. Results of gREMs for *P. pipistrellus* were supported by published data but were ~10 times higher than those of RN‐models. RN‐models use detection/nondetection data, and this loss of information probably affected population estimates of very active species like *P. pipistrellus*.
4. gREM models provided realistic estimates of bat population densities based on automatically recorded call activity data. However, the average flight altitude of species should be accounted for in future analyses. We suggest including flight altitude in the calculation of the detection range to assess the detection sphere more accurately and to obtain more precise density estimates.

## Graphical abstract

Generalized random encounter modeling has been developed to estimate population density from automatically recorded calls. However, this approach has so far not been tested on empirical data. We provide a test of this model on bat calls, prove its value, and suggest a simple but effective modification to improve its accuracy.

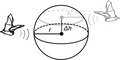

## 1 INTRODUCTION

Automated recording units (ARUs) have become valuable tools to investigate insectivorous bats, which communicate and orientate using acoustic signals. ARUs are commonly used to assess the species composition of different sites (MacSwiney, Clarke, & Racey, 2008), as many species produce distinctive calls. Compared with traditional methods like mist netting, ARUs are noninvasive and detect species that are elusive and difficult to capture (Russo & Voigt, 2016). Moreover, ARUs are cost efficient and can be used to survey large areas over long periods at comparatively little personnel cost. Large‐scale monitoring programs like the North American Bat Monitoring Program benefit from these advantages (Loeb et al., 2015). Furthermore, bats are important indicators for human‐induced changes of the environment (Jones, Jacobs, Kunz, Wilig, & Racey, 2009) and are frequently included in environmental impact assessments. Thus, ARUs have become invaluable tools for determining the presence and quantifying the activity of species in assessment areas.

Bat researchers working with ARUs have used various measures of calling activity. For example, Adams, McGuire, Hooton, and Fenton (2015) used the number of calls, and Suarez‐Rubio, Ille, and Bruckner (2018) used the lengths of call sequences to characterize activity. These measures are appropriate to investigate ecological characteristics of bats, like their habitat preferences. However, they do not provide information about population sizes and dynamics because there is no evidence that bat activity and density relate to each other in any simple and predictable way. This may bear problems in management decisions since activity measures are not regarded of the same value as density in many monitoring programs and environmental impact assessments.

As an example, the European Habitats Directive aims at sustaining and improving the conservation status of bats, among other organisms (European Commission No L 206/9, 1992). Since its announcement in 1992, the directive has become an important standard for many assessment procedures across the EU. It defines the conservation status of a species as favorable if “population dynamics data […] indicate that it is maintaining itself” (European Commission, 1992). Hence, quantifying the density of individuals in a population and the changes of density over time is a prerequisite for justifying protection against potential impacts due to anthropogenic activities on the species included in the Habitats Directive. So, how does one translate data on empirical activity into density estimates?

Two well‐established approaches appear promising at first when it comes to estimating bat population densities from passive acoustic recordings, namely mark–recapture and distance sampling methods. Their application on bats is, however, thwarted by two factors: (a) recorded bat calls cannot be attributed to the vocalizing individuals, a requirement of mark–recapture methods (Marques et al., 2013) and (b) the distance between observer (the recorder) and the recorded animal is unknown, but is necessary in distance sampling methods (Buckland et al., 2001). To circumvent these problems, methods without these preconditions have been applied to estimate population densities of various animal groups. Spatially explicit capture–recapture methods are used to estimate the density of birds (Dawson & Efford, 2009) and anurans (Stevenson et al., 2015), and transect methods for sperm whale densities (Hastie, Swift, Gordon, Slesser, & Turrell, 2003). Both methods deploy arrays of many microphones to record and locate vocalizing animals. But how to deal with data from single recorders, which many practitioners are limited to in monitoring programs and impact assessments? Kloepper et al. (2016) estimate the colony size of emerging cave‐dwelling bats using only one ARU. However, this approach is only useful if all individuals narrowly pass a recording site (the cave entrance) and fly in one direction (out of the cave). In their foraging habitats, bats enter the detection sphere of a recorder from multiple directions. Thus, the Kloepper et al. (2016) method is not applicable in these settings.

Another promising approach to estimate population densities of foraging bats using single ARUs is described by Lucas, Moorcroft, Freeman, Rowcliffe, and Jones (2015). They extend the random encounter models (REMs) often used in camera trap studies (Rowcliffe, Field, Turvey, & Carbone, 2008) to make them suitable for acoustic data (“generalized random encounter models,” referred below as “gREM”). Both REMs and gREMs model the situation of animals entering the detection sphere of a stationary recorder. They require knowledge about a species’ flight speed, signal angle, and call intensity, but do not make assumptions about flight direction and location. However, gREMs simplify the three‐dimensional detection sphere of ARU microphones to two dimensions (Lucas et al., 2015). This might limit their applicability, as the models do not account for individuals entering the detection sphere from above. Despite this constraint, gREMs are currently the most promising method for single ARU data.

In this study, we examined whether gREMs are suitable for estimating population densities of bats using automatically recorded calls. We calculated models using empirical data of three insectivorous bat species with different flying and foraging behaviors: the common pipistrelle *Pipistrellus pipistrellus* (Schreber, 1774), the Northern bat *Eptesicus nilssonii* (Keyserling & Blasius, 1839), and Natterer's bat *Myotis nattereri* (Kuhl, 1817)*.* We mimicked a typical acoustic survey and deployed one recording device at every investigation site for one night. To validate the obtained density estimates, we compared our results to (a) published density estimates based on roost surveys and (b) the output of Royle and Nichols (2003) models that use repeated detection/nondetection data.

## 2 MATERIALS AND METHODS

### 2.1 Study area

The bat vocalization data were recorded in the Harz National park in northern Germany (51°48′N, 10°37′E). The area is located in the transition of the European oceanic and the humid continental climate zone (Belda, Holtanová, Halenka, & Kalvová, 2014), with elevation ranging from 250 to 1,141 m asl (Baumann, 2014). Most of the park's area is covered with Norway spruce *Picea abies* forests. Peat bogs, quarries, small rivers, and two water dams are noteworthy landscape elements within the forest matrix. Three small settlements and two towns of regional importance are located close to the park.

### 2.2 Survey point selection

We distributed 127 sample points over the study area based on a stratified random sampling design. For this, we used an unpublished vegetation map of the national park to define 19 vegetation classes and used them as sampling strata. Since spruce forest dominated the vegetation classes and potentially important minor classes were scattered and covered only small areas, the number of survey points allocated to each class was based on area and the number of GIS polygons. Thus, between 3 and 11 survey points were allocated to each vegetation class.

We sampled bat vocalizations from June to September 2016 and 2017, deploying one ARU per survey point and night. Only nights without rainfall, strong wind, and hunting activity were sampled, and we deployed as many ARUs in parallel as logistically possible. In the first year, 19 points were discarded because of a defect device. Further five points were removed as outliers since excessive activity at those points due to roost vicinity showed a clear effect on the models. In 2017, one point was removed because of forest clear‐cut and two further points due to flooding. Four points were discarded as outliers. Thus, we used data from 103 survey points in 2016 and from 120 survey points in 2017.

### 2.3 Study species

We selected three bat species because they were the most abundant in the surveys and represented different foraging guilds. *Pipistrellus pipistrellus* is an edge space aerial forager (Seibert, Koblitz, Denzinger, & Schnitzler, 2013), *E. nilssonii* an open space forager, and *M. nattereri* an edge space aerial‐hawking forager (Denzinger & Schnitzler, 2013).

### 2.4 Recording and analysis of bat calls

We used 17 automated recording units (ARUs—“batcorder,” ecoObs GmbH, Nürnberg, Germany) of three different generations (1, 2.0, and 3.1). Results derived from different batcorder generations do not differ, as they all housed identical electret microphones (FG series, Knowles Electronics) and were calibrated by the manufacturers prior to each survey year. ARUs were placed at 2.20 m height on metal rods. We used the ARU’s default settings (quality: 20, post‐trigger: 400 ms, threshold level: −27 dB, critical frequency: 16 kHz) and used recordings from sunset to sunrise for the analysis. After placing the ARUs at all sample points, we repeated the process until each point was sampled five times (two times in 2016 from June to mid‐July and from mid‐July to late August; three times in 2017 in June, July, and August).

We used bcAdmin 3.5.6 (ecoObs GmbH) to organize the bat call sequences (passes) and automatically measure their acoustic properties (e.g., main frequency). After that, we used batIdent 1.5 (ecoObs GmbH) to obtain automated identifications of the sequences. We manually validated the batIdent output using two variants since the reliability of automated bat call identification has been strongly questioned (Russo & Voigt, 2016). First, in a strict manner, we either accepted or dismissed the identification output from batIdent and ignored all recordings not attributed to one of the three target species by the software. Secondly, we identified all recorded sequences manually to species level, without reference to the batIdent output*.* In both instances, we used bcAnalyze Pro 1.3.1 (ecoObs GmbH) to inspect vocalizations graphically and the criteria in Hammer and Zahn (2009) and Skiba (2003) to identify species. Thus, we obtained a “strict” data set and a “generous” data set with a lower and higher probability of false‐positive errors, respectively.

### 2.5 Generalized random encounter modeling

Generalized random encounter models (gREMs) are based on the assumption that an acoustic signal needs to cross a hypothetical line *p* within the detection zone of a recorder to be detected (Lucas et al., 2015). The length of *p* depends on the direction of the signal encountering a recorder's detection zone. To account for the different direction possibilities, an averaged $\bar{p}$ is calculated. Calculation of requires different formulae depending on the ARU’s detection angle *θ*, the acoustic signal angle *α,* and the detection distance *r*. The appropriate formula given our assumptions is as follows:

(1) $$\bar{p}=\frac{r}{n}\left(\theta \sin\frac{\alpha }{2}-\cos\frac{\alpha }{2}+1\right)$$

After calculating $\bar{p}$, population density *D* of the vocalizing species can be calculated by:

(2) $$D=z/vt\bar{p}$$

where *z* is the number of detections (=number of recorded sequences between sunset and sunrise), *v* the average speed of the animal, and *t* the recording time in seconds from sunset to sunrise.

Before applying gREMs to calculate $\bar{p}$, it is necessary to know the signal angle of the target species. Although Jakobsen, Ratcliffe, and Surlykke (2013) showed that Vespertilionidae species exhibit similar signal angles independent of their size and weight, divergent figures for different species are reported in the literature. Field studies showed maximal signal angles of 42° for *P. pipistrellus* (Seibert et al., 2013) and 25° for Daubenton's bat *Myotis daubentonii* (Surlykke, Pedersen, & Jakobsen, 2009)*.* A laboratory study with the big brown bat *Eptesicus fuscus* reported maximal angles of 70° (Ghose & Moss, 2003). To account for this discrepancy, we varied *α* for each species using 25°, 42°, and 70°. The detection angle *θ* was set to 200° (V. Runkel, personal communication March 2018).

The detection distance *r* depends mainly on atmospheric and geometric attenuation of sound (Evans, Bass, & Sutherland, 1972). Atmospheric attenuation is influenced by abiotic factors (temperature, air humidity, and atmospheric pressure) and sound characteristics (frequency and sound pressure level; Stilz & Schnitzler, 2012). At each sampling point and night, we recorded temperature using “tinytag2” loggers (Gemini Data Loggers Ltd, Chichester, UK), while relative air humidity data were obtained from four weather stations within the National park. The atmospheric pressure was calculated using the barometric formula (Warnecke, 1997). To characterize call frequency, we used main frequencies, namely 44 kHz for *P. pipistrellus*, 28 kHz for *E. nilssonii*, and 48 kHz for *M. nattereri* (Skiba, 2003)*.* For the sound pressure levels (SPLs), Surlykke and Kalko (2008) reported ~110 to 120 dB for open space and edge space foragers. Since data for edge space aerial‐hawking foragers like *M. nattereri* were not available from literature, the SPL for this species was assumed to be 90 dB, as bats foraging close to vegetation tend to call at lower SPLs (Stilz & Schnitzler, 2012). We used the full range of SPL values as a characterization of uncertainty. Using this approach, we obtained r values of 15.6–40.1 m for *P. pipistrellus*, 27.8–69.3 m for *E. nilssonii,* and 14.1–36.6 m for *M. nattereri*.

After calculating $\bar{p}$, we computed density *D*, which required knowledge about the species’ speed *v*. Seibert et al. (2013) reported average flight speeds of 5.4 m/s (2.5–6 m/s) for *P. pipistrellus*, which was consistent with other authors (Hughes & Rayner, 1993; Schaub & Schnitzler, 2007; Thomas, Jones, Rayner, & Hughes, 1990). Rydell (1993) suggested flight speeds of 4–8 m/s for *E. nilssonii*, which is similar to other bats (Boonman, Parsons, & Jones, 2003). *Myotis nattereri's* speed varied from 1.5 m/s up to 5 m/s but is mainly 4.2 ± 0.9 m/s (Melcon, Denzinger, & Schnitzler, 2007; Siemers & Schnitzler, 2000). Thus, we set *v* at 5.4 m/s for *P. pipistrellus*, 6 m/s for *E. nilssonii,* and 4.2 m/s for *M. nattereri.* We calculated *D* separately for each sampling period and species, using both the strict and generous data set.

### 2.6 Royle–Nichols models

To test the population density estimates obtained with gREMs, we applied Royle–Nichols models (RN‐models) (Royle & Nichols, 2003). RN‐models are based on the assumption that the detection probability at a survey point *p_ij_* depends on the species’ site‐specific abundance *N_i_*:

(3) $$p_{\mathrm{ij}}=1-{\left(1-r_{\mathrm{ij}}\right)}^{\mathrm{Ni}}$$

where *r_ij_* is the detection probability of a single individual. *N_i_* is Poisson distributed with mean *λ_i_*

(4) $$N_{i}∼\text{Poisson}\left(\lambda_{i}\right)$$

Conversely, knowing *p_ij_* allows us to draw conclusions about *N_i_*. Repeated visits at a survey point generate a history of detection/nondetection events *y_ij_*, from which *p_ij_* is estimated. The *y_ij_* are modeled as Bernoulli variables:

(5) $$y_{\mathrm{ij}}|N_{i}∼Bernoulli\left(p_{\mathrm{ij}}\right)$$

To generate a history of multiple detection/nondetection events at each survey point, we handled each recording night as an additive of multiple recording sessions. This was based on the assumption that the studied bats visited their foraging habitats several times during the night (Davidson‐Watts & Jones, 2006; Siemers & Schnitzler, 2000; Smirnov, Vekhnik, Kurmaeva, & Baishev, 2^2013^). In consequence, one individual could be recorded more than once a night. We calculated the number of recording sessions of a night using Scott's rule for estimating optimal bin width of histograms (Scott, 1979). Since night length varied during the surveys, we standardized recording time by dividing the recording time after sunset of a call sequence by total night length. Call activity data within each recording session were transformed to detection/nondetection data.

Covariates which might have influenced detectability were modeled with a logit link function:

(6) $$\text{logit}\left(r_{\mathrm{ij}}\right)=\alpha_{0}+\alpha_{\text{temperature}}+\alpha_{\text{temperature}}^{2}+\alpha_{\text{humidity}}+\alpha_{\text{humidity}}^{2}+\alpha_{\text{slot}}+\alpha_{\text{slot}}^{2}+\alpha_{\text{slot}}^{3}+\alpha_{\text{slot}}^{4}+\alpha_{\text{batcorder}}$$

where *α*_temperature_ is the median temperature during each recording session, *α*_humidity_ the median relative humidity, *α*_slot_ the number of the recording session during a night, and *α*_batcorder_ the ID of the ARU used.

At each survey point, we characterized 73 site‐specific variables (Appendix S1). We selected the covariates with the highest impact on *r* using a random forest analysis (R package party 1.3.3, Hothorn, Buehlmann, Dudoit, Molinaro, & Van Der Laan, 2006; Strobl, Boulesteix, Kneib, Augustin, & Zeileis, 2008; Strobl, Boulesteix, Zeileis, & Hothorn, 2007) and the number of recording sessions with detection events as the response variable. We set the random forest parameter mtry to 18 (approximately twice the square root of the number of variables) and raised parameter ntree until results were stable. When covariates were correlated (Pearson's correlation coefficient >0.6), the variable with lower impact was excluded from subsequent analysis. The covariates selected were modeled with log‐link functions. All continuous covariates were centered on their means.

We fitted models using the occuRN() function of the R package unmarked 0.12.2 (Fiske & Chandler, 2011). First, we chose the best detection model without any site‐specific covariates based on Akaike's information criterion (Akaike, 1987) using the package AICcmodavg 2.2.1 (Mazerolle, 2017). Then, we used that model and included the site‐specific covariates obtained from the random forest to fit a final model (Appendix S2). We applied the MacKenzie & Bailey approach to assess goodness of fit and estimate an overdispersion parameter (MacKenzie & Bailey, 2004). For this, we used Linden, Fuller, Royle, and Hare's (2017) modification of mb.gof.test() in the AICcmodavg package. We calculated 2,000 bootstrap samples for each fit assessment.

We estimated density *D* by dividing the mean *λ* by the median of various minimum convex polygon figures as a measure of home range size obtained from the literature (for *P. pipistrellus:* Davidson‐Watts & Jones, 2006 and Davidson‐Watts, Walls, & Jones, 2006, for *E. nilssonii:* Frafjord, 2013 and Haupt, Menzler, & Schmidt, 2006, for *M. nattereri:* Siemers, Kaipf, & Schnitzler, 1999 and Smith & Racey, 2008)*.* Density was only calculated when models were successfully fitted to the data (nonsignificant chi square value of the goodness of fit statistics, *p* > .05; Appendix S3). Like for the gREMs, we estimated density for each sampling period and species, using both the strict and generous data set of each species. Both the gREM and the RN‐model analyses were conducted in R 3.4.4 (R Core Team, 2018).

We compared the estimates derived from gREM and RN‐models with estimates of *M. nattereri, P. pipistrellus,* and similar‐sized species (Brown Long‐eared bat *Plecotus auritus* and *M. daubentonii*) from Jones, Altringham, and Deaton (1996) and Speakman et al. (1991) which we averaged by their means. For *E. nilssonii,* we used estimates for *M. nattereri* as reference values, because estimates for this species are not available from literature and *M. nattereri* is similar sized to *E. nilssonii*. We expect that population densities are similar in similar‐sized species (Damuth, 1981).

## 3 RESULTS

**Figure 1:**
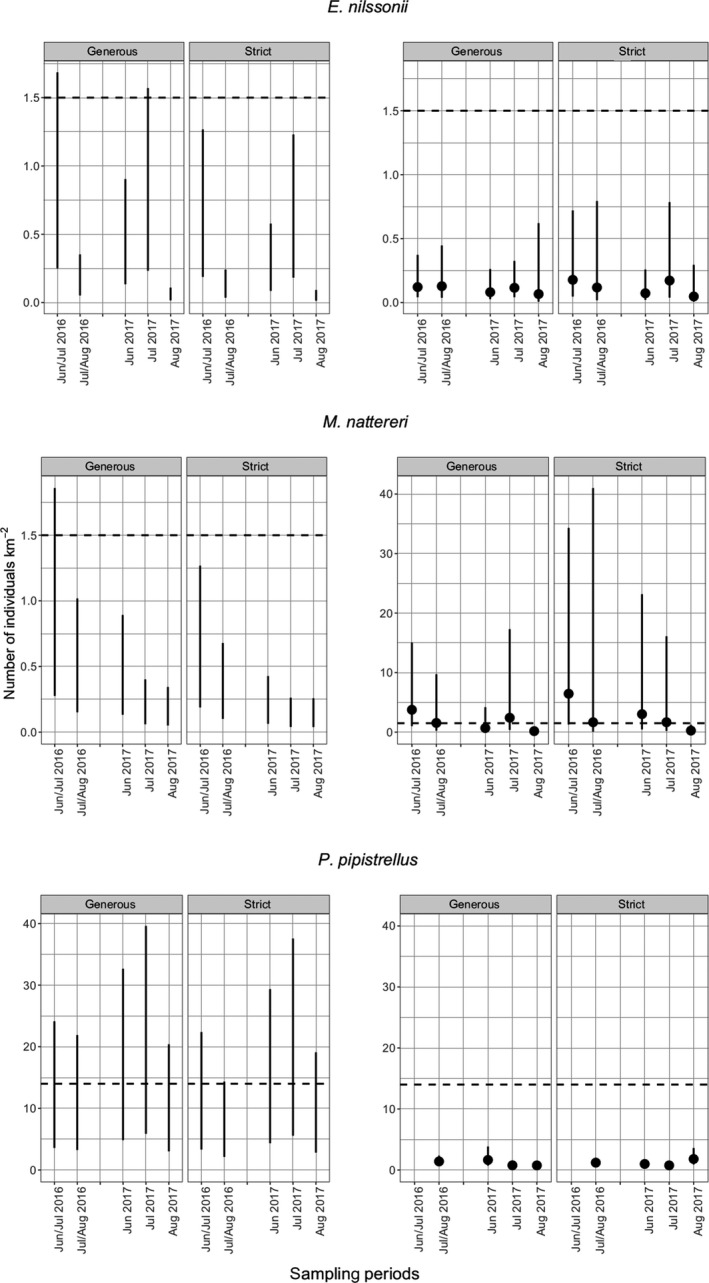
Estimates of population density for *E. nilssonii*, *M. nattereri,* and *P. pipistrellus* based on acoustical data using two different models. Left panels show estimates based on the generalized random encounter models (gREMs). Here, the vertical lines connect calls of 25° and 90 dB to calls of 70° and 120 dB, respectively. Right panels show estimates based on Royle–Nichols models (RN‐models). The dots indicate mean density and the vertical lines represent the 95% confidence intervals. “Generous” and “strict” refer to the different identification variants. The dashed line indicates average estimates derived from literature

We recorded 10,575/9,353 (generous/strict identification) call sequences (passes) of *P. pipistrellus*, while *M. nattereri* (238/153) and *E. nilssonii* (770/569) were less frequent. When applying gREMs, average estimated densities were 0.3/0.2 (generous/strict) individuals of *E. nilssonii* km^‐2^, 0.3/0.3 individuals of *M. nattereri* km^‐2^, and 7.0/5.5 individuals of *P. pipistrellus*/km^2^. The estimates were not affected by identification variants but were highly influenced by the choice of signal angle *α* and sound pressure level SPL (Figure 1).

The density estimates of *E. nilssonii* were generally lower than those reported in the literature. The same was true for *M. nattereri*, but results matched the literature values better if a low detection range was assumed (α = 25°, SPL = 90 dB). Only in *P. pipistrellus*, the bandwidth estimates overlapped with literature values in both sampling periods and identification variants (Figure 1).

The goodness of fit statistics for the RN‐models indicated overdispersion for some of the models (Appendix S3). As it is unclear if estimating the overdispersion parameter $\hat{c}$ using the approach of MacKenzie and Bailey was an appropriate procedure (Linden et al., 2017), we refrained from model adjustments which might have improved $\hat{c}$. Similar to the gREMs, the density estimates based on the strict and generous data sets did not differ greatly (Figure 1).

For *E. nilssonii* and *M. nattereri*, the RN estimates were in agreement with those derived from the gREMs (Figure 1), while for *P. pipistrellus*, the RN estimates amounted to only ~10% of the gREM estimates.

## 4 DISCUSSION

Our results indicate that, in general, generalized random encounter models (gREMs) appropriately estimated population densities of insectivorous bats using automatic stationary recording units. The density estimates were in the same order of magnitude as the two comparison benchmarks. For *M. nattereri* and *P. pipistrellus*, the gREM estimates were comparable to published estimates. For *M. nattereri* and *E. nilssonii*, the estimates matched, in terms of magnitude, those calculated using Royle–Nichols (RN) models.

We used RN‐models as a benchmark against which to compare the gREM estimates. For *M. nattereri* and *E. nilssonii*, the RN results were in agreement with the gREM estimates. Both species were rarely recorded, that is, the number of call sequences per recording session was low. Thus, the information loss inherent in using detection/nondetection data was negligible in these species. In contrast, *P. pipistrellus* exhibited high activity, and the number of call sequences per recording session was often high. Accordingly, the gREM estimates ranged an order of magnitude higher than those of the RN‐models. Therefore, using detection/nondetection data may have biased RN density estimates for highly active bat species.

Studies on other animal groups support our findings for very active species. Comparing population density estimates of fishers *Pekania penannti* using RN‐models and spatial capture/recapture estimates (Linden et al., 2017), and of Siamese Firebacks *Lophura diardi* using RN estimates and distance sampling (Suwanrat, Ngoprasert, Sutherland, Suwanwaree, & Savini, 2015), has demonstrated that RN‐models perform significantly poorer (i.e., underestimation of densities) than the alternative methods. Therefore, RN‐models may be inappropriate for estimating densities of common species using acoustic recordings.

Our data show that gREM results were sensitive to the assumed signal angle *α* and sound pressure level SPL of the calls. The estimates assuming wide *α* and high SPL were up to 10 times lower than those based on narrow angle and low levels. Both average *α* and SPL of a species are closely related to the species’ foraging behavior (Jakobsen, Brinkløv, & Surlykke, 2013) and may explain any deviations of the gREM from the RN‐model results and the literature. For *M. nattereri*, low SPLs better represent this species since it forages near vegetation structures and hence tends to utter soft sounds (Faure, Fullard, & Dawson, 1993). Our density estimates (0.3–1.9 individuals/km^2^) corresponded with the estimates by Jones et al. (1996) who calculated population densities of 1.8 individuals/km^2^ in roosting sites of *M. nattereri* in northern England*.* We also compared our estimates to other similarly sized species: *P. auritus* and *M. daubentonii* (1.4 and 1.0 individuals/km^2^, respectively) (Jones et al., 1996) as population densities of mammals correlate strongly with their body mass (Damuth, 1981). Our estimates were similar to those of Jones et al. (1996) and also to those of Speakman et al. (1991): *P. auritus*: 1.66 individuals/km^2^ and *M. daubentonii*: 2.4 individuals/km^2^.

For *P. pipistrellus*, we expected that estimates based on intermediate SPLs would be most realistic on average (10–15 individuals/km^2^), as this species emits low‐intensity calls when foraging close to vegetation and then switches to higher SPLs in open spaces (Schnitzler & Kalko, 2001). Our estimates were in accordance with findings of Jones et al. (1996) and Speakman et al. (1991) who calculated densities of 12.6 and 18.2 individuals/km^2^, respectively. For *E. nilssonii*, we assumed that an estimate based on a high SPL (0–1.7 individuals/km^2^) was the most plausible because the species is an open space forager and is thus likely to call with high volume (Faure et al., 1993).

In addition to the RN‐models, the literature estimates might underestimate population densities. Estimates which are based on roost surveys should be regarded as minima since roosts are difficult to locate and bats often shift roost locations (Speakman et al., 1991). Nevertheless, estimates from similar‐sized North American species match the European estimates in terms of magnitude (Davis & Hitchcock, 1965; Pearson, Koford, & Pearson, 1962; Twente, 1955). Thus, we are confident that the literature estimates we used to validate gREM results are reliable in terms of magnitude.

**Figure 2:**
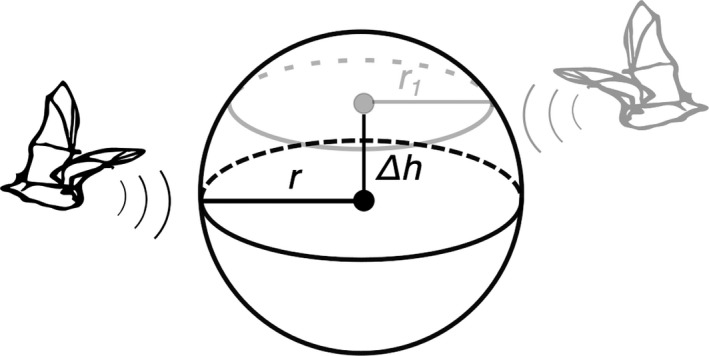
The black bat enters the detection sphere of an automated recorder at the height of the microphone. Its detection range is r, and generalized random encounter models may confidently estimate the density of its population. The gray bat flies higher than the microphone and its detection range *r_1_* is smaller than *r*. To properly estimate densities, the height difference Δ*h* should be accounted for

Although our density estimates matched those found in the literature, the accuracy of gREMs can be improved. In the gREMs, an implicit assumption is that bats vocalize at the level of the microphones, which is unlikely for many recorded call sequences. If a bat enters the detection sphere above the microphone level, the true detection range is shorter than the modeled one (Figure 2). In this case, the detection area of the recorders is larger than assumed, and therefore, density is underestimated. This is especially problematic in species that tend to move at a high flight altitude, like *E. nilssonii* (Rydell, 1993), and may explain the notably lower density estimates compared with similarly sized species.

Given that the detection sphere of a microphone is truly spherical, we suggest including the height difference Δ*h* between the microphone and the average bat flight altitude in Equation 1.

(7) $$\bar{p}=2\sqrt{r^{2}-\Delta h^{2}}\left(\sin\frac{\alpha }{2}\right)$$

This hypothetical detection sphere is still an approximation since a bat emits a cone of sound (Jakobsen, Ratcliffe, et al., 2013). Future research should focus on this complexity of bat calls to further enhance gREMs.

The geometrical solution in Equation 7 would account for the underestimation of the detection area and would improve the accuracy of gREM estimates, especially for species that fly at high altitudes. One may argue, however, that flight altitude is not a constant variable but varies among individuals and landscapes. Indeed, bats adjust their foraging behavior to that of their prey (Krauel, Ratcliffe, Westbrook, & McCracken, 2018; McCracken et al., 2008) and to the spatial configuration of the landscape they are moving in (Roeleke, Bumrungsri, & Voigt, 2018). However, if researchers are successful in representing the landscape heterogeneity when deploying their devices and if many replicate survey points are performed, this variability will be mitigated.

Our results indicate that species behavior can strongly influence gREM density estimates. This is not surprising since both REMs and gREMs are known to vary heavily with the movement speed of individuals and (in gREMs) the call characteristics of species (Lucas et al., 2015; Rowcliffe et al., 2008). Although several researchers have measured the flight speed and vocalization behavior of bats in flight cages (Ghose & Moss, 2003; Seibert et al., 2013), outdoor data under unconfined conditions are scarce. More and better studies on these parameters would render gREM density estimates more accurate and would assure the applicability of gREMs in monitoring programs and impact assessments.

Another aspect to consider when using these models is that both REMs and gREMs assume that animals move randomly with respect to recording units. Although gREMs have not been tested on empirical data, when compared to count data and DNA‐based censuses, REM estimates seem biased (Balestrieri et al., 2016; Cusack et al., 2015). These biases were probably due to the nonrandom behavior of the authors’ study organisms (lion *Panthera leo* and pine marten *Martes martes*, respectively). The precondition of random behavior is also questionable for many bat species that use flight paths to move between their foraging spots (Schaub & Schnitzler, 2007) or exhibit specific habitat preferences (Carr, Zeale, Weatherall, Froidevaux, & Jones, 2018). It should be noted that this nonrandom behavior may affect our density estimates, although we have no evidence for this. Possible biases may have been mitigated by the homogeneous landscape of the investigated area (mostly spruce forests), making pronounced aggregation of activity less likely than in more heterogeneous landscapes. However, animal movement should be considered in future studies using tracking data as a way to mitigate biases caused by nonrandom behavior of animals. This could improve the accuracy of gREMs similar to the spatio‐temporally explicit random encounter model (Jousimo & Ovaskainen, 2016).

## 5 CONCLUSION

Our findings demonstrate that, in contrast to RN‐models, generalized random encounter modeling (gREM) is very promising for estimating bat population densities based on automated recordings of calling activity. We suggest a simple yet effective modification of gREM as a first step for improving its accuracy when the average flight altitude of bats is higher than the level of the recording detectors.

Given that the approaches we used to validate gREM estimates (RN‐models and literature‐based estimates) might underestimate the population density, we are confident that these estimates are reliable, at least in their magnitude. While comparing the gREM estimates to a known animal density would be ideal, this is particularly difficult for bats for which there are essentially no perfectly known populations or well‐proven methods for estimating densities. Therefore, considering the limitations, the comparison made here is a best‐possible comparison.

It is important to note that the biological data needed for setting gREM parameters are scarce, even for the comparatively well‐studied European bat species. We encourage researchers to continue studying the signal angles, sound pressure levels, and flight speed and altitudes of as many species as possible. Moreover, manufacturers should fully provide the microphone characteristics of their products, especially the detection angle, as this is an important variable for gREMs. Those data will help improve the accuracy of future gREM density estimates and promote their applicability in monitoring programs and environmental impact assessments—areas where automated, stationary acoustic detectors show their strengths.

## AUTHORS’ CONTRIBUTIONS

MM and AB conceived the study. AS, MSR, and AB designed the surveys. AS conducted the 2016 fieldwork and quantified the habitat parameters. MM conducted the 2017 fieldwork and analyzed the data. MM and AB lead the writing of the manuscript. All authors discussed the results, critically contributed to the manuscript, and approved the final version.

## Data Availability

The analyzed data are available in the Dryad digital repository: https://doi.org/10.5061/dryad.hx3ffbg9m.
