# Supplementary material for: Estimating population density of insectivorous bats based on stationary acoustic detectors: A case study
Source: Ecol Evol. 2020 Jan 28;10(3):1135–44. doi: 10.1002/ece3.5928 (PMC7029071; doi:10.1002/ece3.5928)
Supplement: Supplementary file 1 [file ECE3-10-1135-s001.pdf]

Appendix S1. Variables, which were used to model site specific detection probability and the abundance using Royle-Nichols models and how they were quantified.

abbreviation	unit	definition	method
tree.age	[a]	average tree age	analysis using ArcGIS 10.2 (ESRI, Redlands, California) based on shapefiles provided by the National park
domin.treespec		dominant tree species	analysis using ArcGIS based on shapefiles provided by the National park
tree.dens		tree density at a sampling point	distance of next tree in each vegetation quadrant to the sampling point, area[m^2]*d[m^{-2}] (area = 100 m^2, d= mean distances to trees) (Mühlenberg 1989)
settlement.dist.tot	[m]	distance of the sampling point to the nearest artificial building	analysis using ArcGIS based on shapefiles provided by the National park
street.all.dist	[m]	distance of the sampling point to the nearest street	analysis using ArcGIS based on shapefiles provided by the National park
street1.dist	[m]	distance of the sampling point to the nearest broad forest path	analysis using ArcGIS based on shapefiles provided by the National park

street2.dist	[m]	distance of the sampling point to the nearest narrow forest path	analysis using ArcGIS based on shapefiles provided by the National park
street3.dist	[m]	distance of the sampling point to the nearest hiking trail	analysis using ArcGIS based on shapefiles provided by the National park
waterbody.dist.tot	[m]	distance of the sampling point to the nearest water body	analysis using ArcGIS based on shapefiles provided by the National park
stream.dist	[m]	distance of the sampling point to the nearest running water body	analysis using ArcGIS based on shapefiles provided by the National park
standwater.dist	[m]	distance of the sampling point to the nearest standing water body inside the National Park	analysis using ArcGIS based on shapefiles provided by the National park
standwater.dist.tot	[m]	distance of the sampling point to the nearest standing water body	analysis using ArcGIS based on shapefiles provided by the National park
stream.leng.buf30	[m]	length of running water bodies within a radius of 30 m around the sampling point	analysis using ArcGIS based on shapefiles provided by the National park
stream.leng.buf85	[m]	length of running water bodies within a radius of 85 m around the sampling point	analysis using ArcGIS based on shapefiles provided by the National park
stream.leng.buf140	[m]	length of running water bodies within a radius of 140 m around the sampling point	analysis using ArcGIS based on shapefiles provided by the National park

stream.leng.buf200	[m]	length of running water bodies within a radius of 200 m around the sampling point	analysis using ArcGIS based on shapefiles provided by the National park
street.all.leng.buf30	[m]	length of paths/trails within a radius of 30 m around the sampling point	analysis using ArcGIS based on shapefiles provided by the National park
street.all.leng.buf85	[m]	length of paths/trails within a radius of 85 m around the sampling point	analysis using ArcGIS based on shapefiles provided by the National park
street.all.leng.buf140	[m]	length of paths/trails within a radius of 140 m around the sampling point	analysis using ArcGIS based on shapefiles provided by the National park
street.all.leng.buf200	[m]	length of paths/trails within a radius of 200 m around the sampling point	analysis using ArcGIS based on shapefiles provided by the National park
street1.leng.buf30	[m]	length of broad forest paths within a radius of 30 m around the sampling point	analysis using ArcGIS based on shapefiles provided by the National park
street1.leng.buf85	[m]	length of broad forest paths within a radius of 85 m around the sampling point	analysis using ArcGIS based on shapefiles provided by the National park
street1.leng.buf140	[m]	length of broad forest paths within a radius of 140 m around the sampling point	analysis using ArcGIS based on shapefiles provided by the National park
street1.leng.buf200	[m]	length of broad forest paths within a radius of 200 m around the sampling point	analysis using ArcGIS based on shapefiles provided by the National park

street2.leng.buf30	[m]	length of narrow forest paths within a radius of 30 m around the sampling point	analysis using ArcGIS based on shapefiles provided by the National park
street2.leng.buf85	[m]	length of narrow forest paths within a radius of 85 m around the sampling point	analysis using ArcGIS based on shapefiles provided by the National park
street2.leng.buf140	[m]	length of narrow forest paths within a radius of 140 m around the sampling point	analysis using ArcGIS based on shapefiles provided by the National park
street2.leng.buf200	[m]	length of narrow forest paths within a radius of 200 m around the sampling point	analysis using ArcGIS based on shapefiles provided by the National park
street3.leng.buf30	[m]	length of hiking trails within a radius of 30 m around the sampling point	analysis using ArcGIS based on shapefiles provided by the National park
street3.leng.buf85	[m]	length of hiking trails within a radius of 85 m around the sampling point	analysis using ArcGIS based on shapefiles provided by the National park
street3.leng.buf140	[m]	length of hiking trails within a radius of 140 m around the sampling point	analysis using ArcGIS based on shapefiles provided by the National park
street3.leng.buf200	[m]	length of hiking trails within a radius of 200 m around the sampling point	analysis using ArcGIS based on shapefiles provided by the National park
altitude	[m]	altitude above sea level	analysis using ArcGIS based on shapefiles provided by the National park
canopy.cover	[%]	canopy coverage at the sampling point	picture shot with fisheye lens

zonation.park		management zones of the National park (development zone or core zone) at the sampling point	GIS data provided by the National park
tree.height.max	[m]	highest tree within the vegetation cross	highest tree within the vegetation cross
tree.dbh.max	[cm]	tree with maximal diameter at breast height within the vegetation cross	maximal diameter at breast height of four trees per quadrant
tree.height.med	[m]	median of tree heights within the vegetation cross	median of highest trees per quadrant
tree.dbh.med	[cm]	median of dbh within the vegetation cross	median of dbh of four trees per quadrant
snags		standing dead wood within the vegetation cross	maximum of standing dead wood per quadrant, scale: 0(0 dead trees), 1(1-2 dead trees), 2(3-5 dead trees), 3(5-10 dead trees), 4(>15 dead trees)
heatsum	[°C]	sum of temperature at one m height within a night at the sampling point	Tinytag Plus 2 data logger
veg.cover0_0.5		vegetation cover at 0-0.5 m within the vegetation cross	mean of presence-absence of vegetation at 13 marks within the vegetation cross
veg.cover0.5_1		vegetation cover at 0.5-one m within the vegetation cross	mean of presence-absence of vegetation at 13 marks within the vegetation cross
veg.cover1_1.5		vegetation cover at one-1.5 m within the vegetation cross	mean of presence-absence of vegetation at 13 marks within the vegetation cross

veg.cover1.5_2	vegetation cover at 1.5-two m within the vegetation cross	mean of presence-absence of vegetation at 13 marks within the vegetation cross
veg.cover2_3	vegetation cover at two - three m within the vegetation cross	mean of presence-absence of vegetation at 13 marks within the vegetation cross
veg.cover3_5	vegetation cover at three - five m within the vegetation cross	mean of presence-absence of vegetation at 13 marks within the vegetation cross
veg.cover5_7	vegetation cover at five - seven m within the vegetation cross	mean of presence-absence of vegetation at 13 marks within the vegetation cross
veg.cover7_10	vegetation cover at seven - ten m within the vegetation cross	mean of presence-absence of vegetation at 13 marks within the vegetation cross
veg.cover10_15	vegetation cover at ten -15 m within the vegetation cross	mean of presence-absence of vegetation at 13 marks within the vegetation cross
veg.cover>15	vegetation cover at more than 15 m within the vegetation cross	mean of presence-absence of vegetation at 13 marks within the vegetation cross
patch.divers.buf30	diversity of vegetation classes within a radius of 30 m around the sampling point	FRAGSTATS 4.2.1 (McGarigal, Cushman, & Ene, 2012) (Simpson´s Diversity Index)
patch.divers.buf85	diversity of vegetation classes within a radius of 85 m around the sampling point	FRAGSTATS (Simpson´s Diversity Index)
patch.divers.buf140	diversity of vegetation classes within a radius of 140 m	FRAGSTATS (Simpson´s Diversity Index)

		around the sampling point	
patch.divers.buf200		diversity of vegetation classes within a radius of 200 m around the sampling point	FRAGSTATS (Simpson´s Diversity Index)
patch.totedge.buf30	[m]	length of vegetation class borders within a radius of 30 m around the sampling point	FRAGSTATS (Total Edge)
patch.totedge.buf85	[m]	length of vegetation class borders within a radius of 85 m around the sampling point	FRAGSTATS (Total Edge)
patch.totedge.buf140	[m]	length of vegetation class borders within a radius of 140 m around the sampling point	FRAGSTATS (Total Edge)
patch.totedge.buf200	[m]	length of vegetation class borders within a radius of 200 m around the sampling point	FRAGSTATS (Total Edge)
veg.type		vegetation class at the sampling point	GIS data provided by the National park
patch.no.buf30		number of vegetation classes within a radius of 30 m around the sampling point	FRAGSTATS (Number of Patches)
patch.no.buf85		number of vegetation classes within a radius of 85 m around the sampling point	FRAGSTATS (Number of Patches)
patch.no.buf140		number of vegetation classes within a radius of 140 m around the sampling point	FRAGSTATS (Number of Patches)
patch.no.buf200		number of vegetation classes within a radius of 200 m around the sampling point	FRAGSTATS (Number of Patches)
patch.rich.buf30		richness of vegetation classes within a radius of 30 m around the sampling point	FRAGSTATS (Patch Richness)
patch.rich.buf85		richness of vegetation classes within a radius of 85 m around the sampling point	FRAGSTATS (Patch Richness)
patch.rich.buf140		richness of vegetation classes within a radius of 140 m around the sampling point	FRAGSTATS (Patch Richness)
patch.rich.buf200		richness of vegetation classes within a radius of 200 m around the sampling point	FRAGSTATS (Patch Richness)
patch.rich%.buf30	[%]	relative patch richness within a radius of 30 m around the sampling point	FRAGSTATS (Relative Patch Richness)
patch.rich%.buf85	[%]	relative patch richness within a radius of 85 m around the sampling point	FRAGSTATS (Relative Patch Richness)
patch.rich%.buf140	[%]	relative patch richness within a radius of 140 m around the sampling point	FRAGSTATS (Relative Patch Richness)

patch.rich%.buf200	[%]	relative patch richness within a radius of 200 m around the sampling point	FRAGSTATS (Relative Patch Richness)
mig.mean		measure of structural complexity based on images	imagemetrics 1.0 (Massicotte, 2014), rgdal 1.4.4 (Bivand, Keitt, & Rowlingson, 2018), raster 2.8.18 (Hijmans, 2017)
