# Supplementary material for: Estimating population density of insectivorous bats based on stationary acoustic detectors: A case study
Source: Ecol Evol. 2020 Jan 28;10(3):1135–44. doi: 10.1002/ece3.5928 (PMC7029071; doi:10.1002/ece3.5928)
Supplement: Supplementary file 2 [file ECE3-10-1135-s002.pdf]

Appendix S2. Best Royle-Nichols models according to AIC. r_{ij} is the detection probability at a sampling point and visit, λ_i is the site specific abundance. We refer to Appendix S2. For explanations of covariates.

M. nattereri:

sampling period	data set	model
Jun-Jul 2016	generous	$\text{logit}(r_{ij}) = \alpha_{temp}{}^2 + \beta_{humidity}$
		$\log(\lambda_i) = \beta_{patch.divers.buf85} + \beta_{stream.leng.buf30}$
	strict	$\text{logit}(r_{ij}) = \alpha_{humidity}{}^3 + \beta_{temp}{}^2$
		$\log(\lambda_i) = \beta_{patch.divers.buf85}$
Jul-Aug 2016	generous	$\text{logit}(r_{ij}) = \alpha_{humidity}{}^3$
		$\log(\lambda_i) = \beta_0$
	strict	$\text{logit}(r_{ij}) = \alpha_{temp} + \beta_{humidity}{}^2$
		$\log(\lambda_i) = \beta_{street2.leng.buf200}$
Jun 2017	generous	$\text{logit}(r_{ij}) = \alpha_0$
		$\log(\lambda_i) = \beta_{veg.cover1.5\_2} + \beta_{tree.dbh.med} + \beta_{veg.totedge.buf200}$
	strict	$\text{logit}(r_{ij}) = \alpha_0$
		$\log(\lambda_i) = \beta_{veg.cover1.5\_2} + \beta_{veg.totedge.buf200}$
Jul 2017	generous	$\text{logit}(r_{ij}) = \alpha_0$
		$\log(\lambda_i) = \beta_{snags} + \beta_{veg.cover.tot} + \beta_{street2.leng.buf200}$
	strict	$\text{logit}(r_{ij}) = \alpha_0$
		$\log(\lambda_i) = \beta_{veg.cover0.5\_1} + \beta_{street2.leng.buf200}$
Aug 2017	generous	$\text{logit}(r_{ij}) = \alpha_{temp} + \beta_{humidity}{}^2$
		$\log(\lambda_i) = \beta_{altitude} + \beta_{veg.cover0.5\_1}$
	strict	$\text{logit}(r_{ij}) = \alpha_{rel.hum}$
		$\log(\lambda_i) = \beta_{tree.dens} + \beta_{standwater.dist.tot}$

E. nilssonii:

sampling period	data set	model
Jun-Jul 2016	generous	$\text{logit}(r_{ij}) = \alpha_{\text{temperature}} + \alpha_{\text{humidity}}{}^2$
		$\log(\lambda_i) = \beta_{\text{veg.cover.tot}} + \beta_{\text{settlement.dist}} + \beta_{\text{altitude}} + \beta_{\text{zonation.park}}$
	strict	$\text{logit}(r_{ij}) = \alpha_{\text{temperature}} + \alpha_{\text{humidity}}{}^2$
		$\log(\lambda_i) = \beta_{\text{veg.cover.tot}} + \beta_{\text{settlement.dist}} + \beta_{\text{veg.cover.0\_0.5}} + \beta_{\text{altitude}} + \beta_{\text{zonation.park}}$
Jul-Aug 2016	generous	$\text{logit}(r_{ij}) = \alpha_{\text{slot}}$
		$\log(\lambda_i) = \beta_{\text{tree.height.max}} + \beta_{\text{street3.leng.buf85}}$
	strict	$\text{logit}(r_{ij}) = \alpha_{\text{slot}}$
		$\log(\lambda_i) = \beta_{\text{tree.height.max}} + \beta_{\text{street3.leng.buf85}} + \beta_{\text{street2.dist}}$
Jun 2017	generous	$\text{logit}(r_{ij}) = \alpha_0$
		$\log(\lambda_i) = \beta_{\text{zonation.park}} + \beta_{\text{altitude}}$
	strict	$\text{logit}(r_{ij}) = \alpha_0$
		$\log(\lambda_i) = \beta_{\text{veg.cover0.5\_1}} + \beta_{\text{altitude}}$
Jul 2017	generous	$\text{logit}(r_{ij}) = \alpha_{\text{humidity}} + \alpha_{\text{slot}}$
		$\log(\lambda_i) = \beta_{\text{altitude}} + \beta_{\text{street3.leng.buf140}} + \beta_{\text{tree.height.med}}$
	strict	$\text{logit}(r_{ij}) = \alpha_{\text{humidity}}{}^2 + \alpha_{\text{slot}}$
		$\log(\lambda_i) = \beta_{\text{altitude}} + \beta_{\text{street3.leng.buf140}} + \beta_{\text{veg.cover10\_15}} + \beta_{\text{veg.cover1\_1.5}}$
Aug 2017	generous	$\text{logit}(r_{ij}) = \alpha_{\text{humidity}}$
		$\log(\lambda_i) = \beta_{\text{veg.cover.tot}}$
	strict	$\text{logit}(r_{ij}) = \alpha_{\text{humidity}}$
		$\log(\lambda_i) = \beta_{\text{street1.leng.buf200}}$

P. pipistrellus:

year/period	data set	model
Jun-Jul 2016	generous	$\text{logit}(r_{ij}) = \alpha_{\text{temperature}}^2 + \alpha_{\text{slot}}^4$
		$\log(\lambda_i) = \beta_{\text{snags}} + \beta_{\text{veg.cover3\_5}} + \beta_{\text{settlement.dist}} + \beta_{\text{heatsum}}$
	strict	$\text{logit}(r_{ij}) = \alpha_{\text{temperature}}^2 + \alpha_{\text{humidity}} + \alpha_{\text{slot}}^4$
		$\log(\lambda_i) = \beta_{\text{snags}} + \beta_{\text{veg.cover2\_3}} + \beta_{\text{settlement.dist}} + \beta_{\text{patch.divers.buf30}}$
Jul-Aug 2016	generous	$\text{logit}(r_{ij}) = \alpha_{\text{slot}}$
		$\log(\lambda_i) = \beta_{\text{tree.dens}} + \beta_{\text{veg.cover5\_7}} + \beta_{\text{stream.leng.buf85}} + \beta_{\text{patch.no.buf30}} + \beta_{\text{street3.leng.buf85}} + \beta_{\text{mig.mean}}$
	strict	$\text{logit}(r_{ij}) = \alpha_{\text{slot}}$
		$\log(\lambda_i) = \beta_{\text{tree.dens}} + \beta_{\text{veg.cover5\_7}} + \beta_{\text{stream.leng.buf85}} + \beta_{\text{patch.no.buf30}} + \beta_{\text{patch.divers.buf200}} + \beta_{\text{street3.leng.buf85}} + \beta_{\text{mig.mean}}$
Jun 2017	generous	$\text{logit}(r_{ij}) = \alpha_{\text{temperature}}^2 + \alpha_{\text{humidity}} + \alpha_{\text{slot}}^4$
		$\log(\lambda_i) = \beta_{\text{heatsum}} + \beta_{\text{snags}} + \beta_{\text{street2.leng.buf200}} + \beta_{\text{veg.totedge.buf140}}$
	strict	$\text{logit}(r_{ij}) = \alpha_{\text{temperature}}^2 + \alpha_{\text{slot}}^4 + \alpha_{\text{humidity}}$
		$\log(\lambda_i) = \beta_{\text{heatsum}} + \beta_{\text{veg.totedge.buf140}} + \beta_{\text{street.leng.buf85}}$
Jul 2017	generous	$\text{logit}(r_{ij}) = \alpha_{\text{temperature}}^2 + \alpha_{\text{humidity}} + \alpha_{\text{slot}}^4$
		$\log(\lambda_i) = \beta_{\text{street3.leng.buf85}} + \beta_{\text{stream.leng.buf140}} + \beta_{\text{standwater.dist.tot}} + \beta_{\text{tree.height.med}}$
	strict	$\text{logit}(r_{ij}) = \alpha_{\text{temperature}}^2 + \alpha_{\text{humidity}} + \alpha_{\text{slot}}^4$
		$\log(\lambda_i) = \beta_{\text{street3.leng.buf85}} + \beta_{\text{stream.leng.buf200}} + \beta_{\text{standwater.dist.tot}} + \beta_{\text{tree.height.med}}$
Aug 2017	generous	$\text{logit}(r_{ij}) = \alpha_{\text{temperature}}^2 + \alpha_{\text{humidity}} + \alpha_{\text{slot}}^2$
		$\log(\lambda_i) = \beta_{\text{stream.leng.buf200}} + \beta_{\text{veg.type}} + \beta_{\text{patch.rich.buf30}}$
	strict	$\text{logit}(r_{ij}) = \alpha_{\text{temperature}}^2 + \alpha_{\text{slot}}$
		$\log(\lambda_i) = \beta_{\text{stream.leng.buf200}} + \beta_{\text{veg.type}} + \beta_{\text{patch.rich.buf30}}$
