# Supplementary material for: Estimating population density of insectivorous bats based on stationary acoustic detectors: A case study
Source: Ecol Evol. 2020 Jan 28;10(3):1135–44. doi: 10.1002/ece3.5928 (PMC7029071; doi:10.1002/ece3.5928)
Supplement: Supplementary file 3 [file ECE3-10-1135-s003.pdf]

Appendix S3. Predicted mean abundance per site λ, standard errors (SE) and results of the Chi-squared statistics ($\hat{c}$ and p-value). The asterisks indicate significance.

M. nattereri:

sampling period	generous				strict			
	Predicted λ	SE	$\hat{c}$	p	Predicted λ	SE	$\hat{c}$	p
Jun/ Jul 2016	3.19	2.2	0.52	0.68	5.49	4.59	1.36	0.19
Jul/ Aug 2016	1.31	1.23	1.08	0.19	1.41	2.31	0.49	0.37
June 2017	0.58	0.53	0.35	0.78	2.57	2.53	0.21	0.8
Jul 2017	2.05	2.01	1.02	0.29	1.41	1.57	0.72	0.43
Aug 2017	0.14	0.09	1.26	0.13	0.21	0.15	0.56	0.44

E. nilssonii:

sampling period	generous				strict			
	Predicted λ	SE	$\hat{c}$	p	Predicted λ	SE	$\hat{c}$	p
Jun/ Jul 2016	0.58	0.32	1.34	0.13	0.85	0.59	0.94	0.49
Jul/ Aug 2016	0.61	0.39	0.54	0.79	0.57	0.55	0.77	0.41
June 2017	0.39	0.23	1.61	0.08	0.35	0.22	0.96	0.21
Jul 2017	0.55	0.29	0.40	0.42	0.83	0.63	0.76	0.32
Aug 2017	0.32	0.36	0.17	0.65	0.23	0.21	0.24	0.58

P. pipistrellus:

sampling period	generous				strict			
	Predicted λ	SE	$\hat{c}$	p	Predicted λ	SE	$\hat{c}$	p
Jun/ Jul 2016	3.19	2.2	2.12	0.02*	5.49	4.59	1.93	0.02*
Jul/ Aug 2016	1.31	1.23	0.91	0.61	1.41	2.31	1.13	0.15
June 2017	0.58	0.53	1.34	0.07	2.57	2.53	1.1	0.22
Jul 2017	2.05	2.01	0.48	0.96	1.41	1.57	0.52	0.92
Aug 2017	0.14	0.09	0.05	0.41	0.21	0.15	0.92	0.87
