# Supplementary material for: Estimating population density of insectivorous bats based on stationary acoustic detectors: A case study
Source: Ecol Evol. 2020 Jan 28;10(3):1135–44. doi: 10.1002/ece3.5928 (PMC7029071; doi:10.1002/ece3.5928)
Supplement: Supplementary file 4 [file ECE3-10-1135-s004.pdf]

The following abstract was submitted to the 176th meeting of the Acoustical Society of America. After the presentation of the study, the abstract was published in The Journal of the Acoustical Society of America (for details, see below).

# Estimating population densities of temperate, insectivorous bats based on automatically recorded calls

The Journal of the Acoustical Society of America 144, 1817 (2018);
https://doi.org/10.1121/1.5068014

Estimating population density based on automatically recorded calls is a key topic in bioacoustics, since individual recognition of animals is impossible. Several recently developed models do not require individual recognition but nevertheless allow to estimate density. However, there is a need to test these models on empirical data. Here, we used generalized random encounter models (gREM) to estimate population densities based on automatically recorded bat calls. To check the validity of the derived estimates, we fit Royle-Nichols models to species detection/non-detection data. Estimates of the two models were compared to each other and to estimates from published studies.

The estimates of both models and literature estimates were within the same order of magnitude.

Both models give reliable estimates of population density. However, we provide some cautionary notes for practical use: Bats which enter the detection sphere from above might bias results of gREMs, as the model simplifies the detection sphere to a two-dimensional area. On the other hand, reduction to detection/non-detection data in Royle-Nichols models results in information loss, which could limit their applicability in common species.

Finally, we recommend to consider species behaviour carefully when applying one of the tested models.
